# Smell and taste disorders: warning signs for SARS-CoV-2 infection^☆^

**DOI:** 10.1016/j.bjorl.2020.05.003

**Published:** 2020-05-30

**Authors:** Klinger Vagner Teixeira da Costa, Aline Tenório Lins Carnaúba

**Affiliations:** a Universidade Federal de Alagoas (UFAL), Maceió, AL, Brazil; b Centro Universitário Cesmac, Maceió, AL, Brazil

Brazil, the second most populous country in the Americas, faces a public health crisis with more than 66,000 confirmed cases and 5000 deaths from COVID-19. Due to the worldwide demand for tests to identify patients infected with SARS-CoV-2, health strategies are severely hindered by the lack of tests that can guide the correct and immediate isolation of positive cases of COVID-19. In the last few weeks, it was observed that the loss of smell/taste associated with COVID-19 has been a frequent complaint, mainly identified when evaluating the patient with appropriate tools. However, how useful are these symptoms in clinical practice, and especially in the absence of tests for SARS-CoV-2?

In a recent study, Menni et al.^1^ showed that the combination of loss of smell/taste, fever, cough, was predictive for a positive test for COVID-19, with sensitivity of 0.54 (0.44; 0.63), specificity of 0.86 (0.80; 0.90) and, overall, the loss of smell/taste had a positive predictive value of 61.7%.

A multicenter European study also showed that anosmia and ageusia were independently and strongly associated with a positive COVID-19 test (anosmia: aOR = 10.9; 95% CI: 5.08–23.5; ageusia: aOR = 10, 2; 95% CI: 4.74–22.1).^2^ In Brazil, without sufficient tests for a massive assessment of the population, especially in regions with few diagnostic and poor resources, the recommendation to isolate a patient with anosmia/ageusia complaint, starting from the medical consultation itself, has become a health strategy to try to control the spread of the virus.

In fact, the loss of smell/taste does not depend on nasal obstruction/rhinorrhea and can begin even before the typical signs/symptoms of COVID-19, thus becoming warning signs even in oligosymptomatic patients and, especially, in the initial stage of the disease, when high viral replication and transmissibility occur. The recovery of smell/taste, when it occurs, usually does so in the first two weeks after the resolution of COVID-19.^3^ However, new interpretations appear every week, and we believe that valuing and actively questioning the patient about olfactory/gustatory disorders can help, implemented not only by otorhinolaryngologists, but the entire health team working on the front line of the pandemic control.

## Conflicts of interest

The authors declare no conflicts of interest.
